# One Page in the History of Starvation and Refeeding

**DOI:** 10.5041/RMMJ.10524

**Published:** 2024-04-28

**Authors:** Deborah E.-S. Hemstreet, George M. Weisz

**Affiliations:** 1 English Communications Coordinator, Rambam Health Care Campus, Haifa, Israel; 2 Editorial Assistant, Rambam Maimonides Medical Journal, Haifa, Israel; 3 School of Humanities, University of New South Wales, Sydney, Australia; 4 School of Humanities, University of New England, Armidale, Australia

**Keywords:** Hunger disease, nutritional therapy, refeeding, starvation, sudden death

## Abstract

There is a long history of starvation, including reports dated back to antiquity. Despite exceptional scientific developments, starvation still exists today. The medical aspects of starvation were well established in the twentieth century, particularly following studies related to the 1943–1944 Bengal famine in India and starved prisoners of war and survivors of World War 2. The refeeding of the starved victims provided disappointing results. Nevertheless, those studies eventually led to the development of a new branch of research in medicine and to the definition of what is now known as refeeding syndrome. This paper briefly reviews the history and groundwork that led to today’s understanding of starvation and refeeding, with a particular emphasis on the observations from studies on starved Holocaust survivors and prisoners of war after World War 2. The relevance of these studies for modern times is briefly discussed.

## INTRODUCTION

Famine and starvation have been recorded throughout the annals of human history. Nevertheless, deaths due to famine during the twentieth century were unique. Researcher Stephen Devereux points out that more than 70 million people suffered famine-related deaths between 1903 and the late 1990s.^1^ In his eyes, their deaths were inexcusable in light of the abundance of food produced during that period. Indeed, between 1900 and 1940, more than 20 famines are mentioned.^2^ However, the extent of starvation experienced during World War 2 (WW2) shocked the Allied forces, who found starving populations in Nazi-subdued nations and emaciated survivors of years of starvation in the Nazi extermination and labor camps. Starvation had been used as a weapon of extermination, a tool subsequently ruled as genocide against humanity by the United Nations.^3^ Prisoners of war (POWs) were also suffering from the effects of prolonged starvation.

Hence, a concentrated effort was made to bring medical care and food to all those affected by starvation. However, contrary to expectations, when rescued Holocaust survivors were reintroduced to rapid nutrition, they sickened and died. In the beginning, many physicians were stymied by the survivors’ failure to recover.^4^ However, subsequent research led to a clinical understanding of refeeding syndrome.

Today, refeeding syndrome is recognized as a condition that can occur following refeeding in malnourished individuals who have experienced no food intake or a pronounced reduction of food intake for at least five days.^5^ Clinically, it is diagnosed by the onset of hypophosphatemia within 2–5 days of refeeding and is accompanied by hypokalemia and/or hypomagnesemia. This electrolyte imbalance can lead to central and peripheral edema, as well as complications affecting the neuromuscular, cardiac, and central nervous systems, including tachycardia, impaired cognition, and ultimately death.

While there is much literature looking at the risks with refeeding, little mention is made of the possible interactions between refeeding and the underlying pathologies caused by starvation, commonly referred to in the literature as “hunger disease.”^6^ Tragically, in light of worldwide events, an awareness of the negative affect of refeeding is essential when caring for survivors of long-term starvation or malnutrition.

This review attempts to provide a comprehensive look at the historical *observations* regarding the recovery (or lack thereof) of starvation survivors and the research that indicated serious consequences following refeeding. Relevance for modern medical practitioners is briefly discussed.

## REFEEDING IN ANCIENT HISTORY

The refeeding problem may have been discussed in antiquity. Hippocrates is frequently cited as having written the first possible description of refeeding syndrome. Hence it merits presentation herein:

… if a person goes seven days without eating or drinking anything, in this period most die; some even survive that time, but still die; and others in fact are persuaded not to starve themselves to death but to eat and drink: however the cavity no longer admits anything because the jejunum has grown together in that many days, and these people too die.^7^^(p161)^

However, Hippocrates considered the problem to be an intestinal obstruction rather than a problem of refeeding. Furthermore, the above-cited text appears in the midst of a discussion related to abortion and fetal viability, unrelated to an extended period of starvation.^7^^(pp161–3)^

Hence, the first authentic description of the risk of refeeding after long-term sub-nutrition can be safely attributed to the historian, Flavius Josephus (Ben Matityahu) (37–100 CE). In his writings regarding the fall of Jerusalem at the hands of the Romans, he wrote that while some fought against the invaders or committed self-destruction, the worst fate was to be a deserter among the Romans:

… for when they came first to the Romans, they were puffed up by the famine, and swelled like men in a dropsy; after which they all on the sudden overfilled those bodies that were before empty, and so burst asunder, excepting such only as were skillful enough to restrain their appetites, and by degrees took in their food into bodies unaccustomed thereto.^8^

The next known lay description of refeeding problems after long-term starvation appears in 1033 by the French monk Rodulfus Glaber (980–1046, known as Rodulfi Glabi). Observing the Burgundy famine, Glaber wrote in his Latin book, *Historianum Libri Quinque*, of the affected population: “even when they received food, they became distended and died immediately.”^9^ Glaber’s description could be attributed to heart failure or hunger edema; these and sudden death upon refeeding are recognized clinical features of refeeding syndrome.^5^,^10^,^11^

The first medical description of what may well be refeeding syndrome was by the Florentine physician Antonio di Paolo Benivieni (born in 1443), in a book published in 1507, some 5 years after his death. Of noble origin, he became the physician to the famous families in Renaissance Florence, including the first three well-known Medici, i.e. Cosimo, Piero Gottoso, and Lorenzo the Magnificent. After performing numerous legally permitted autopsies, Benivieni’s observations on the famine of 1496 described death for a variety of reasons, including starvation, ingestion of toxic food, excessive ingestion after prolonged and forced abstinence from food in adults, and the death of breast-fed children and their starved mothers who ate to satiety.^10^^(p1411)^

A recent publication by Kano et al. describes the deaths of survivors of the “Battle for Tottori Castle” in Japan in 1581.^12^ The siege to take Tottori Castle was 3 months in duration; part of the battle tactic was the deliberate starvation of the soldiers and civilians living therein. Based on a detailed analysis of the documentation from that time, the authors conclude that the high percentage (>50%) of survivors who died soon after being fed was, in all probability, due to refeeding syndrome.

Despite the above observations and the plethora of famines since the 1500s, the cause of death in starved individuals who were rapidly reintroduced to normal nutrition remained a mystery. Nevertheless, many anecdotal recommendations have been passed down advising that food should be eaten cautiously and in small amounts after fasting.^9^

It is worth noting that other than the above items, little could be found in the literature at hand related to refeeding until the twentieth century. At the end of WW2, physicians noted that starved survivors seemed to experience a slower-than-expected recovery upon refeeding, but they remained puzzled as to why.^13^ Problems were also noted in refeeding attempts later in the century on patients with anorexia^14^ and incarcerated individuals on hunger strikes.^15^ Today, although the clinical significance of refeeding syndrome for undernourished patients suffering from a number of medical conditions is better understood, it is still often overlooked.^16^,^17^

## WORLD WAR 2 AND STARVATION

There is nothing ambiguous about starvation. Quite simply, it is a caloric intake well below that needed to remain alive. Starvation is woven through history as a tragic consequence of disease, forces of nature, and the cruelty of war. Medical experts differ with regard to how long one must be starved before death becomes inevitable. This is because, as Hoffer and colleagues point out, there are different *kinds* of starvation.^18^ Hence, mortality varies depending on the extent of undernutrition, both in duration and amount (for example, no food intake for a few days versus a few weeks, and limited intake over a short period of time versus chronic undernutrition). Unlike prolonged severe malnutrition, symptoms related to starvation resulting from medical conditions are further confounded by the underlying disease.

### The Bengal Famine

Hunger and starvation marked the beginning of the twentieth century, with blockades leading to mass starvation during World War 1, the Bengal Famine (1943–1944), and the weaponization of starvation by the Nazis via their notorious “Hunger Plan.”^3^,^19^ Hence, the effects of starvation became a topic of high interest during WW2. Studies emanating from WW2 provided varying degrees of scientific data covering malnutrition, sub-nutrition, starvation, and inanition.

A 1944 report emerged from field experience in Calcutta^20^ and helped set the groundwork for treating victims of starvation.^21^ The authors generally defined three different types of starvation: inanition due to starvation alone; inanition due to combined starvation and disease; and acute disease with relatively little inanition. They provided detailed recommendations for treatment based on inanition type, and introduced the successful use of protein hydrolysates for severe inanition, along with detailed notes for treating common coexisting conditions found in their patients (e.g. anemia, skin conditions, edema, pneumonia, and intestinal disorders). By 1945, there was a general understanding that starvation led to progressive destruction of the digestive, absorptive, and protective functions of the alimentary canal, along with indications of impaired metabolic function.^22^ It was also believed that, despite treatment, end-stage starvation (severe inanition) was the precursor to death. Hence, the British Allies placed great hope on the Calcutta research results when anticipating the starved populations liberated in Europe and from the Nazi camps—in particular Bergen-Belsen.^22^

Nevertheless, much remained unknown regarding the pathophysiology of starvation itself.

### The Minnesota Starvation Experiment

The Allies knew they would encounter an unprecedented number of starved people when liberating Europe. In an attempt to develop an effective plan to rehabilitate the starved masses, the renowned Minnesota Starvation Experiment began in 1944, with initial results published in 1945.^23^ The study recruited 36 male volunteers who underwent controlled starvation for 24 weeks after an initial 12-week baseline control phase. The participants were then “rehabilitated” with an initial restricted refeeding phase followed by unrestricted access to food.

The Minnesota Starvation Experiment represents the only longitudinal study conducted on starvation and rehabilitation. It remains important from a clinical and psychological perspective; however, it could not anticipate the actual conditions encountered by the Allies when they came upon starved masses in Nazi camps, and later on in repatriated POWs. Individuals had undergone years of gross starvation, in some cases subsisting on diets of 400–800 calories per day or less.^3^,^6^ Unlike their European counterparts, the Minnesota Starvation Experiment participants were on a semi-starvation diet of 1600–1800 calories, and they lost no more than 25% of their original body weight. Data for what happened under more severe conditions, to the point of death, would have to come from elsewhere.

### The Warsaw Ghetto Research and the Defining of Hunger Disease

During WW2, several highly trained and knowledgeable physicians were incarcerated in the Warsaw Ghetto. They were faced with an intolerable situation: caring for people who, sooner or later, would die of starvation. An understanding of initial starvation studies from World War 1 and their own recognition of multiple pathologies related to starvation led to a research project that remains relevant today: research to develop a comprehensive clinical and biochemical understanding of severe inanition. The physicians had access to a unique patient population—individuals who had been severely malnourished for months to years. Dr Emil Apfelbaum-Kowalski, one of the researchers, wrote that, in comparison to contemporary studies on the topic, “hunger was never so prolonged as to lead to cachexia and death, the clinical material was never so numerous.”^6^^(p144)^ After the war their research was published and provided a name for the multiple clinical, biochemical, and histological effects of starvation: hunger disease.^6^ Modern scholars may well take issue with certain ethical elements of the Warsaw Ghetto research; however, Janczewska points out that this dilemma does not negate the importance of the Warsaw Ghetto research findings.^24^

Under the leadership of Dr Israel Milejkowski, the researchers carefully screened their patients for medical conditions other than hunger disease.^6^^(pp75–88)^ Patients suffering only from severe malnutrition were included in their study (*n*=100). Malnourished patients suffering from other debilitating diseases were excluded. Of particular relevance were the observations made by the Warsaw Ghetto physicians regarding refeeding. It should be noted that the researchers did not initiate refeeding *per se*. However, they performed experiments that could technically be viewed as refeeding, since they involved the administration of glucose, proteins, and carbohydrates for glucose tolerance tests and to examine the basic metabolic rate in sudden and high amounts, as compared to the diet the patients were receiving. They recorded a poor response to refeeding: as starvation-induced metabolic changes rapidly reversed, the patients’ circulatory systems took longer to adapt, further burdening an already weakened heart, and leading to heart failure and death.^6^ Had their data been available immediately after the war, it could have made an invaluable contribution to the rehabilitation of victims of starvation. Nevertheless, their research is the only cross-sectional study of the unwilling victims of “pure” starvation via severe sub-nutrition over an extended period of time (months to years) and the resultant clinical presentation of hunger disease.

## THE LIBERATION AND DISCOVERY OF A REFEEDING PROBLEM

Following the liberation, no one anticipated the infernal conditions found in the liberated Nazi labor and death camps: not the Red Army that liberated Majdanek and Auschwitz in January 1945, not the British when liberating Bergen-Belsen (March 1945), nor the Americans when liberating Mauthausen (March–April 1945). What they found was filmed and reported worldwide. Journalists, authors, and medical practitioners cited conditions that, in the words of some, even Dante would not have been able to describe.^25^ A former prisoner stated that the liberating armies came upon “the foulest and vilest spot that ever soiled the surface of this earth.”^26^^(p28)^

That the incarcerated had faced a level of starvation beyond comprehension was clear. Their fate upon refeeding, however, was equally horrifying. British soldiers, seeing the walking skeletons, offered their own rich food and chocolate bars. One witness stated: “… the internees simply could not take the rich food and eating it invariably made their condition worse or caused fatalities.”^26^^(p26)^ This was the first report indicative of the dangers of refeeding.

An army reporter wrote that food had to be prepared such that it would not further burden the survivors’ intestines, since otherwise the food seemed to cause intolerable diarrhea and possibly even death.^27^

Within days of being liberated, a substantial number of survivors of the Nazi camps perished. Precise numbers were not recorded, but it is estimated that nearly a quarter of those liberated from Bergen-Belsen died within a few days; the death rate was even higher at other camps.^28^ The connection was made that immediate death occurred within minutes or hours of high caloric refeeding.^29^ Similar events were mentioned when other camps were liberated in Europe, such as the Ebensee Concentration Camp.^30^

Early on, Dr Mark Dvorjetsky, a survivor of 10 different Nazi camps, repeatedly observed deaths related to cardiopulmonary and immune system failure, particularly in Holocaust survivors. The deaths were unexplainable—he believed many of the patients should have survived. Many of his observations fit the classic symptoms of refeeding syndrome.^4^,^31^,^32^

One of the most highly documented camps run by the Nazis was Bergen-Belsen. Originally established as a prisoner of war camp, it was expanded to include a work camp of some 20,000 detainees. In 1944, as the Allies pressed deeper into Germany, the Nazis began transporting prisoners from other camps into Bergen-Belsen. When the British entered, on April 15, 1945, there were around 60,000 prisoners in Bergen-Belsen, most of whom were starving and ill with typhus and tuberculosis.^33^,^34^ According to Ya’akov Lazovik, a past director of the Yad Vashem archives in Jerusalem, Israel, the number of prisoners who died during the first few days after liberation was higher than generally believed – at more than 30%.^35^ In the eyes of the medical community, it is difficult to attribute all these deaths to refeeding, since no precise medical records exist describing the circumstances leading to death.^35^

The physiology of hunger took no sides. Many accounts written by Holocaust survivors, historians, and journalists in the literature describe similar events of death soon after refeeding, regardless of the geographical location.^36^ During the Battle of Stalingrad, by December of 1942, starving German soldiers were provided with food supplements by the German military command. The soldiers died for no apparent reason, after having eaten small amounts of meat paste with a high fat content.^37^,^38^ Anna Reid likewise describes sudden death from refeeding in her account of the siege of Leningrad.^39^ In the comprehensive nine-volume work on the Nazi camp system, *Der Ort des Terror* [The Place of Terror], there was no consensus regarding the number of deaths due to refeeding. The authors made it a point to reject feeding as the cause of the immediate deaths of the survivors and cited pre-existent pathologies as the cause of death in the survivors.^40^

## SELECTED STUDIES ON THE REHABILITATION OF STARVED CIVILIANS, POWs, AND HOLOCAUST SURVIVORS

Several medical publications and documents were eventually published by contemporary researchers on the conditions of Holocaust survivors and POWs at liberation and the implemented feeding policies. One of the first medical reports that discussed death after refeeding during WW2 appeared in *The Lancet* in 1945.^41^ Dutch medical authorities focused on the impact of the 5-month siege under the Nazi occupation that began in November 1944. This was the first medical document on starvation and refeeding that included children, and the second time that the effects on bone metabolism leading to osteoporosis were mentioned.^41^ Their study was the only one to describe the different types of observed death (discussed below), although no hard data were provided.

Shortly thereafter, Collis published a description of the conditions found in Bergen-Belsen. He felt there was no doubt that dried milk and a protein hydrolysate and glucose mixture resulted “in the saving of thousands of lives.” However, again, no hard data supported this statement.^42^

Other publications quickly followed. In the *British Medical Journal*, Captain P.D. Mollison described starvation, laboratory diagnoses, hypoproteinemia, anemia, and the presence of potentially fatal typhus and tuberculosis. He also reported on blood tests and respiratory and renal function investigations.^43^ Similar topics were discussed and published on India’s POWs held in Japanese camps^44^ and on Japanese soldiers who hid in the Philippine mountains and suffered from starvation.^13^

However, although many of these reports are often cited as being among the first to note the presence of refeeding syndrome, none of them overtly recognized rapid refeeding as the defining culprit in the deaths of their patients. Instead, observers and scientists implicated pre-existing conditions, the presence of parasites, and even vitamin deficiencies. Only much later was an electrolyte imbalance theorized. Despite multiple descriptions of symptoms affecting the neuromuscular, cardiac, and central nervous systems, and the near-immediate deaths reported from several liberated camps from both sides of the warring parties and continents, it would take several years to confirm the connection to an electrolyte balance.

On May 29, 1945, a discussion was held by the Royal Academy of Science on the topic of the “Physiology and Treatment of Starvation,” in response to the large number of civilians, survivors of the Nazi camps, and POWs suffering from long-term starvation.^22^ Much hope had been placed on the use of protein hydrolysates based on lessons learned during the Bengal Famine (1942–1944). There had been a certain amount of success in India using protein hydrolysates and a food supplement of flour enriched with sugar, salt, yeast, and shark liver oil, which became known as the Bengal Famine Mixture.^21^ Hence, the discussion focused primarily on parenteral and oral administration of protein hydrolysates and a strictly regimented system of refeeding. The assumption at the time was that the terminal stage of starvation was marked by uncontrollable diarrhea, progressive dehydration, and the inability of the gastrointestinal system to absorb food or fluid.^22^^(p388)^ Dr Janet Vaughan and colleagues reported on their work in Bergen-Belsen at the invitation of the US Army. They found that, in general, parenteral protein hydrolysates were handled better than oral ones, and that dried milk was also well tolerated.^45^ The discussion did not consider “normal” feeding following starvation, nor did it mention that the Bengal Famine Mixture, which had been mass-produced for the starving survivors, was rejected by most who tasted it: it was too sweet for the European palates.^21^,^34^,^46^,^47^ There was general agreement that the physiological damage to metabolic function and the gastrointestinal system made standard food absorption difficult, if not impossible. Overall, the report reflects the medical community’s great disappointment with the failure of protein hydrolysates as an effective nutrition regimen for starved survivors.^45^ Subsequently, Vaughan admitted that “administering *small* amounts of food had been the best treatment for the starved patients.”^47^^(location652)^ However, again, no information was provided pointing to how she came to that conclusion.

Table 1 summarizes the selected study findings for starved survivors of WW2, Nazi extermination/ work camps, and starved POWs, including Japanese survivors of starvation. While the clinical findings align well with hunger disease, other data may point to additional medical issues. These studies helped lay the groundwork for our understanding of starvation and provided indications that refeeding was indeed a problem.

**Table 1:** Summary of Selected Studies of Starved Holocaust Survivors and Prisoners of War, with Added Commentary in Brackets.

Reference	Study Group	Clinical Findings	Nutritional Rehabilitation and Results	Study Outcomes and Observations
Burger et al. (1945)^41^	Post-liberation population of Western Holland (children and adults)	Anemia (Hgb ~11 g/dL) with tendency to leukopenia Bodily and mental exhaustion Body temperature ≤35°C Chemosis Diarrhea [assumed due to past infection but no pathogens detected] Dizziness Edema in minority of pts* Emaciation Heart: normal but bradycardia (avg. 40 bpm) and low systolic BP (80 mmHg) [cardiac decompensation in some pts*] Osteoporosis with bone pain† Slightly reddened tongue	Acid casein hydrolysate (5% IV): caused thrombosis; no positive results after administration (treatment discontinued) Enzymic protein hydrolysates: little effect on edema, adynamia, apathy; no benefit in children; most benefit for pts unable to swallow or eat due to mental disturbances All pts: high-caloric diet encouraged Pts with high-protein and high-caloric diet recovered best [no data provided on how many pts died at beginning of feeding]	Complications included cardiac decompensation [possibly indicative of electrolyte imbalance, but no other data provided] Three types of deaths: (1) sudden, unexplained, early after hospital admission; (2) unexpected following apparent recovery; (3) slow death after lapsing into a coma* Autopsies: bronchopneumonia in most cases; some atrophied livers, hearts, and spleens* Results of plasma or whole blood infusions “not encouraging”^41^^(p283)^ Reddened or painful tongue responded well to nicotinic acid or nicotinamide injection, respectively [possible indicator of vitamin B3 deficiency, known to occur in refeeding syndrome] [No mortality data or causes of death listed; no electrolyte studies mentioned]
Collis (1945)^42^	Survivors of Belsen (preliminary report)	Dysentery Starvation (pure starvation cases presented with edema, gingivitis, pigmentation (?pellagra), emaciation, and extreme lassitude) Tuberculosis Typhus	Supervised special feedings (not defined) Protein hydrolysate and glucose, but refused by many pts due to fear of torture Dried milk	Reduced death rate of 300/day to 60/day after a few weeks; attributed to improved nutrition [Very general report with little medical data]
Vaughan et al. (1945)^45^	Bergen-Belsen survivors—representative group of pts (small numbers)	Edema (gross or general) Diarrhea Low plasma protein Starvation Pellagra-like tongue lesions [indicative of vitamin B3 deficiency] [Primary focus of study was response to hydrolysates]	Oral hydrolysates: Nasal drip (2 pts), poorly tolerated; 1 pt died on d2; second pt worsened condition [Death and poor recovery may have been related to sudden increased nutritional supplementation] Oral, only slight improvement; 1 pt developed colicky pain and ascites but improved on milk diet	All pts receiving milk improved well Most stool cultures negative Blood volume tests planned but could not be performed General conclusion: oral hydrolysates not helpful in starvation pts; pts “suffered from many intercurrent infections”^45^^(p397)^ [not confirmed by testing] [No hard data provided; no mention of results of vitamin supplementation]
Mitchell and Black (1946)^48^	Mostly British and Eastern European POWs from Japanese camps	Diarrhea or dysentery Emaciation Malaria Malnutrition Normal heart (altered heart sounds) Weight loss: average 41 lb/person In malnutrition only pts Edema, sometimes ↑ on recovery Slow, shallow respirations Generally feeble pulse AND unexplained tachycardia in extreme malnutrition cases Neurological signs Reddened tongue [possible sign of vitamin B3 deficiency] Vitamin deficiencies (riboflavin and nicotinic acid)	Vitamin supplements Five-stage (S) graduated diet: S1, protein hydrolysate 7× for 1d; S2, protein hydrolysate + egg-milk mixture 7× per day until appetite returns; S3, egg-milk mixture + rolled oats, tinned fruit, tinned chicken for at least 3d; S4, hospital light diet; S5, hospital ordinary diet Generally good response and improvement of physical condition (e.g. less diarrhea and edema) Signs of vitamin deficiency usually only after refeeding [See also Study Outcomes and Observations]	23 deaths between Sept. 9 and Nov. 30, 1945 Most deaths related to malnutrition and other underlying cause (e.g. beriberi, tuberculosis) Sudden increase in diet a contributing cause in at least one death, possibly others Relapses in health condition consistently responded well to further reduction in calories Autopsy findings (most pts): small internal organs, atrophic stomach and intestinal mucosa, and pulmonary edema [might indicate CHF due to electrolyte imbalance indicative of refeeding syndrome; electrolytes were not examined] “Careful dieting and individual feeding were the only effectual measures in treating the most severe cases of malnutrition.”^48^^(p862)^
Mollison (1946)^43^	Bergen-Belsen survivors (exams performed on a selected group of children and adult starvation survivors)	Anemia (normocytic) ↓ Blood volume Diarrhea Edema Emaciation Hypoproteinemia Low BP (avg 91/60) Normal heart (reduced intensity sounds) Tachycardia (avg >100 bpm) Tuberculosis (40% of those examined)/typhus (most pts) Weight loss (29%–56% of original weight in those strong enough to be weighed)	“Enough food in terms of calories, but much of it was unpalatable”^43^^(p5)^ Survivors refused liquid diets, preferred solid food Some survivors feared eating due to immediate and severe diarrhea [No details regarding diets or vitamin supplementation]	Frequent deaths observed: thinnest pts died first; cause of death assumed to be secondary to tuberculosis in many of the cases evaluated Unsatisfactory recovery correlated to tuberculosis [No other details provided]
Walters et al. (1947)^44^	Indian POWs held by the Japanese, ~2000 pts in 4 groups§	All groups: anemia in varying severities (macrocytic); delayed edema, hypocalcemia, hypoproteinemia G1 (60%): wasting only—slight anemia G2 (~1%): wasting + severe hypoproteinemia; anemia (severe); blood and plasma volume (low); edema with ascites (severe); wasting (extreme) G3 (10%): wasting and vitamin deficiency; riboflavin or nicotinic acid symptoms, often seen together G4 (~30%): wasting and neurological syndrome—peripheral neuritis due to beriberi (20%); “captivity cord syndrome” (2%); “captivity amblyopia” (9%) In general: sub-normal blood pressure	All groups: graduated diet from low-residue/bland food (~3800 calories) when appetite was low to a full diet of 5300 calories, high-protein, with high-caloric and high-protein diets	G1–G3: Good recovery G4: Good recovery except for most severe cases In pts with diarrhea, increased food intake aggravated the condition Indications that Indian people more susceptible to macrocytic anemia; captivity syndrome seemed to be unique to prisoners held in the Middle or Far East; diarrhea not necessarily part of starvation syndrome Phosphorus and calcium levels were comparable to normal controls in 27 representative pts, but albumin was very low [No mention of deaths]
Schnitker et al. (1951)^13^	Japanese POWs Out of 8000 pts, a representative group of 24 pts studied‡ G1: Massive edema (*n*=12) G2: None or minimal edema (*n*=12) Controls: 24 apparently healthy Japanese males for comparison	Anorexia Creatinuria Diarrhea (evidence of poor intestinal absorption) Dyspnea Edema Hydrothorax and ascites (G1 only) Hypoproteinemia Intestinal parasites (all pts) Liver function tests abnormal (all pts) Malaria Pleural effusion and congestion (G1 only) Tachycardia on exertion Vitamin deficiency rare, indications of vitamin A deficiency present Weakness Weight loss (~40% of original weight)	All pts: high-calorie, high-vitamin diet with yeast and vitamin supplements	Slow response to treatment despite high-caloric diet and vitamin supplementation Poor tolerance for large amounts of food presumed due to intestinal lesions [autopsy found no intestinal lesions in pts] Signs of gastrointestinal disturbances 5 pts in study group died (data available for only 4); 6 other malnutrition cases also autopsied; presumed cause of death was starvation Autopsies: subcutaneous fat scant or absent, skeletal muscles grossly atrophic, atrophy of heart and pancreas (*n*=5); atrophied liver (*n*=4); atrophic cirrhosis of liver (*n*=1); intestinal ulcers (*n*=3; bacterial cause confirmed in only 1 pt) Noticeably few pts with signs of vitamin deficiency despite neurologic changes observed [indicative of vitamin B3 deficiency] No explanation for massive edema in some pts vs none in others
Winick (1979)^6^	Hospitalized pts in the Warsaw Ghetto 100 pts suffering only from hunger disease	General weakness Tiredness Reduced physical effort Somnolence attributed to hypoglycemia	All pts immediately moved from starvation diet (less than 1000 cal/day) to 1000-calorie diet Glucose tolerance tests subjected starved pts to sudden sugar load Basic metabolic rate tests subjected subjects to sudden protein/ carbohydrate load via eggs	Following sugar loading, hypoglycemia <60 mg% common, reaching levels as low as 32 mg% Low Cl(−) levels observed in blood, gastric juice and urine Low urine Cl(−) levels associated with edema

Studies are presented in the order of publication.

* Precise data not provided.

† “Some pts with starvation.”^41^^(p282)^

‡ This was an observational study of the results of standard medical care and a good diet for severe starvation in its various stages and manifestations. No special interventions on the study group were allowed under the rules of the Geneva Convention (the study group had to receive the same care, diet, and treatment as the other pts).

§ The authors estimated the percentages.

BP, blood pressure; bpm, beats per minute; CHF, congestive heart failure; d, day; G1, group 1; G2, group 2; G3, group 3; G4, group 4; POWs, prisoners of war; pt(s), patient(s).

## OVERVIEW OF RECORDED DEATHS OF SURVIVORS

The anecdotal and clinical observations detailed herein describe three types of deaths due to starvation, which Burger et al. specifically detailed.^41^ These were:

- slow death after lapsing into a coma,
- unexpected death following apparent recovery; and
- sudden, unexplained death early after hospital admission.

While the slow deaths might have been inevitable, it was the unexpected deaths that most puzzled the authors of these studies.

### Slow Death

Slow deaths usually occurred after the patient had lapsed into a coma. Autopsies revealed bronchopneumonia and varying degrees of atrophy to the liver, spleen, kidneys, and heart, all of which are related to chronic semi-starvation.^6^ All the autopsied Japanese POWs had bronchopneumonia and atrophied hearts and livers^13^ and were comparable to the autopsy results of Burger et al.’s data on starved civilians in Western Holland^41^ as well as the autopsy data emanating from the Warsaw Ghetto.^6^ Given the condition of these patients, their recovery was highly unlikely and their deaths were probably due to end-stage starvation and other underlying conditions.

### Unexpected Deaths after Refeeding or Hospital Admission

To this day, the actual cause of unexpected deaths (sudden or delayed) after refeeding remains unknown. However, as previously noted, the unique conditions surrounding these fatalities prompted speculation that the immediacy of the death after eating might have been erroneously reported, or that the deaths were due to another concurrent underlying condition coinciding with refeeding.^13^,^40^

Nevertheless, the abundant observational accounts of unexpected deaths within minutes to hours after starving civilians, Holocaust survivors, and POWs consumed food cannot be ignored.

The literature includes many instances of delayed deaths after refeeding. As pointed out above, it is difficult to state unequivocally that these deaths were related to refeeding due to the lack of data. However, mineral metabolism and vitamin imbalances were noted, both of which are recognized elements of refeeding syndrome.^37^,^49^ Several of the observations in the WW2 studies included hypophosphatemia, hypokalemia, hypomagnesemia, hyponatremia, hypocalcemia, and certain vitamin deficiencies (see Table 1).

In refeeding syndrome, these imbalances can be corrected within 4–5 days with the appropriate treatment; however, treatment must be administered quickly, in conjunction with appropriate measures to stabilize the cardiovascular system.^49^ Failure to do so leads to cardiac decompensation, cardiac arrest, and death^37^,^50^—precisely the causes of death that puzzled Dr Dvorjetsky.^31^,^32^

### Possible Pathophysiologies Contributing to Unexpected Deaths

There is no doubt that the physical condition of those who died unexpectedly was precarious; they too may have been quite near to end-stage starvation, and other issues may have contributed to these unexpected deaths. Patients suffering from starvation manifest in different ways. All exhibit wasting. However, some but not all became edematous or developed neurologic defects. Refeeding may have only been one component contributing to their deaths, but it could also have been the proverbial straw that broke the camel’s back.

The patients in the WW2 studies were diagnosed with a number of maladies. While many patients were reintroduced to food on a graduated diet, dietary rehabilitation generally focused on a high caloric intake.^13^,^41^,^44^,^48^ Looking more closely at possible pathologies in the patients studied may give important pointers to the role refeeding might have played in those patients who died unexpectedly.

#### Anemia

Anemia seems to have affected all patients in the WW2 studies; however, the Indian soldiers captured by the Japanese showed a high prevalence of macrocytic anemia. 44 In contrast, other studies (in a primarily European population) revealed normocytic anemia.^41^,^43^

The Warsaw researchers were certain that hunger anemia was not due to increased destruction of the red blood cells nor to the increased volume of circulating blood found in their patients. Rather, it was primarily due to the lack of “all kinds of food” and that the only effective therapy was “proper diet.”^6^^(p186)^ Indeed, with the exception of patients given blood transfusions for anemia,^41^ adjunct therapies with different types of iron supplements did not seem to help the malnourished survivors of WW2.^41^,^43^,^44^ Rather, the anemia improved slowly over time, coinciding with improved dietary intake.^41^,^43^,^44^ In individuals with an already failing heart due to hypophosphatemia and volume overload, despite the clear comments of some researchers that patients’ hearts were normal,^41^,^43^,^48^ anemia could have been an additional factor leading to unexpected death.

#### Intestinal disorders

Diarrhea was prevalent in nearly all survivors examined. However, some researchers disagreed with the assumption that diarrhea was part of hunger disease.^44^ Infectious causes of diarrhea were quite common; hence they cannot be ruled out. Intestinal lesions, which can cause diarrhea, can develop as a result of severe and long-term starvation. The issue of intestinal damage had been discussed at length in the 1945 meeting of the Royal Academy of Science.^22^ There were numerous reports of people experiencing uncontrollable diarrhea to the point of incontinence after refeeding.^19^,^43^,^44^,^47^ Autopsies of the Japanese POWs confirmed the presence of colonic ulcers, but not in all.^13^ Likewise, the Warsaw Ghetto research found intestinal changes in only 27.2% of those autopsied. Hence, it seems that intestinal lesions were not the primary cause of sudden death in Holocaust survivors and POWs. Contrary to the assumptions of the Royal Academy of Science discussion, intestinal lesions were not a common component of hunger disease.^6^

#### Vitamin deficiencies

Most of the WW2 studies found only minimal vitamin deficiencies. Nevertheless, a majority of starved patients presented with slightly reddened tongues and mouths, which many thought to be indicative of a nicotinic acid (vitamin B3) deficiency.^22^,^48^ Burger et al. observed that patients with reddened or painful tongues responded well to nicotinic acid.^41^ Vitamin B3 deficiency may help explain the diarrhea noted in all patients and may have been one aspect of the neurological symptoms noted in Japanese prisoners of war^13^ and POWs incarcerated by the Japanese.^44^,^48^

Acute thiamine (vitamin B1) deficiency is known to occur in refeeding syndrome.^18^ The Warsaw researchers noted indications of thiamine deficiency and wondered if it was related to hunger edema.^6^^(p104)^ Mitchell and Black routinely administered both nicotinic acid and thiamine to the recovering POWs.^48^ While high thiamine doses generally helped patient rehabilitation, they noted 58 malnutrition cases presenting with unexplained tachycardia, unresponsive to thiamine administration. The authors remained puzzled as to the cause of the tachycardia.

Walters and colleagues noted a high number of neuropathies in rehabilitating Indian POWs who had been held by the Japanese.^44^ They felt this was indicative of multiple vitamin deficiencies, including vitamin A, vitamin B1, and vitamin B3 (nicotinic acid). However, while high vitamin doses helped many of the recovering POWs, they failed to help many others. It should be noted that while all the POWs had been placed on graduated diets, the starting caloric intake was rather high (~3,800 cal/day).^44^ Schnitker et al. noted similar issues in a study group consisting of Japanese POWs (*n*=24).^13^ All their patients were also on high-caloric diets.

Vitamin B1 seems to have also been administered to some of the above-mentioned patients. There are indications that refeeding with carbohydrates can increase cellular thiamine utilization, leading to acute thiamine deficiency.^49^ This may have occurred in at least a few of the patients discussed above.

#### Heart failure

The status of the cardiovascular system in hunger disease is particularly interesting when comparing the WW2 studies with the wealth of data gleaned by the Warsaw Ghetto researchers despite their grim circumstances.

Much remains unknown. However, the Warsaw Ghetto researchers noted that, in hunger disease, cardiac failure was due to inadequate blood supply to the organs rather than the passive congestion found in other types of heart failure.^6^^(p152)^ They noted that their patients suffering from hunger cachexia had low-voltage electrocardiogram tracings^6^^(p146–7)^ with low-amplitude T-waves; the ST segments were commonly depressed. They also demonstrated that most patients with hunger cachexia had an increased blood volume per kilogram of weight.^6^^(p134)^ To accommodate these changes, the heart rate decreased, resulting in reduced cardiac output. Unlike more nourished individuals, in hunger disease patients there were minimal increases in systolic and mean pressures after physical exertion, with the heart rate remaining unchanged. The Warsaw Ghetto studies indicated that following prolonged starvation, the atrophying heart could adapt, but only to a point. Beyond a certain threshold, the heart was unable to increase cardiac output in response to *any* increased demand, leading to an imbalance between the body’s hemodynamic and metabolic needs.

Physical examination in most of the WW2 studies revealed no significant cardiac pathologies other than bradycardia^41^ or tachycardia,^13^,^43^,^48^ and reduced-intensity heart sounds^43^,^48^ in severely starved survivors. However, autopsies revealed atrophied hearts in nearly all those with a history of starvation, in addition to other atrophied organs.^6^,^13^,^41^ At autopsy, Mitchell and Black noted that all internal organs were smaller than normal, not just the heart, and they recorded the presence of pulmonary edema,^48^ indicative of congestive heart failure. Similar to Mitchell and Black, Mollison noted the presence of tachycardia and reduced-intensity heart sounds in Bergen-Belsen survivors.^43^ Burger et al., on the other hand, noted bradycardia.^41^ This raises an important question: since bradycardia is known to be associated with hunger disease, why was tachycardia detected in so many of the starved Holocaust survivors and POWs? Given the study description, it is highly likely that the POWs and Bergen-Belsen survivors were examined only after refeeding had commenced. Their hearts may not have reached the advanced end-stage observed in patients with hunger cachexia, as documented in the Warsaw Ghetto studies. Still, cardiac compensatory mechanisms may not have been enough to meet the increased metabolic needs of Mitchell and Black’s and Mollison’s patients. If so, then the tachycardia might have been an early sign of refeeding syndrome, indicative of fluid overload and cardiac failure.^37^

Hence, in general, the hearts of patients in the WW2 studies might not have reached the advanced stage observed in patients with hunger cachexia, as documented in the studies from the Warsaw Ghetto.

#### Bronchopneumonia

Both bronchopneumonia (found in many autopsied patients [Table 1]) and wet beriberi (suspected but not confirmed in some patients^13^) will exacerbate cardiac failure and congestive heart failure, leading to death. The bronchopneumonia found on autopsy was probably secondary to the debilitated state of the starved individuals.

#### Metabolic imbalances: electrolytes and hypoglycemia

Hunger disease patients may present with hypophosphatemia, hypomagnesemia, and hypokalemia in varying degrees, depending on the degree of starvation experienced.^17^ However, detection of these conditions may be difficult due to the body’s adaptive mechanism from carbohydrate to fat metabolism when stressed by starvation, which can mask symptoms associated with these imbalances. During prolonged fasting, although serum levels are normal, the body may become depleted of phosphate, magnesium, and potassium.

Hypophosphatemia, hypomagnesemia, and hypokalemia will eventually manifest during refeeding. In response to carbohydrate ingestion, insulin is secreted, and glucagon secretion is halted. Insulin promotes glycogen synthesis, lipogenesis, and protein synthesis. These processes, in turn, shift phosphate, potassium, and magnesium from the extracellular compartment into the cells. Severe hypophosphatemia is associated with muscle weakness and respiratory insufficiency.^51^ Profound hypokalemia may lead to arrhythmias and even cardiac arrest. Hypomagnesemia may lead to cardiac dysfunction and will exacerbate hypokalemia. Increased serum glucose and insulin promote water and sodium retention, leading to volume overload and possible cardiac failure. Increased serum glucose during refeeding will also increase thiamine consumption in favor of carbohydrate metabolism, leading to thiamine deficiency and Wernicke–Korsakoff syndrome.

For a variety of reasons, the electrolyte levels were not measured in most of the WW2 studies. Walters et al. noted low calcium blood levels (8.5 mg/100 mL), although the patients did not present with clinical signs of calcium deficiency. Nevertheless, some of the X-rays they obtained revealed markedly reduced bone densities. In light of unremarkable inorganic phosphorus and serum phosphatase levels, the authors were fairly certain the calcium deficiency was due to malnutrition.^44^

The Warsaw Ghetto researchers likewise noted low calcium levels in pure hunger disease patients, as well as high potassium levels. While they were unable to examine other electrolytes, their research led them to conclude that uncompensated metabolic acidosis was at work in hunger disease.^6^^(p94)^

**Figure 1:**
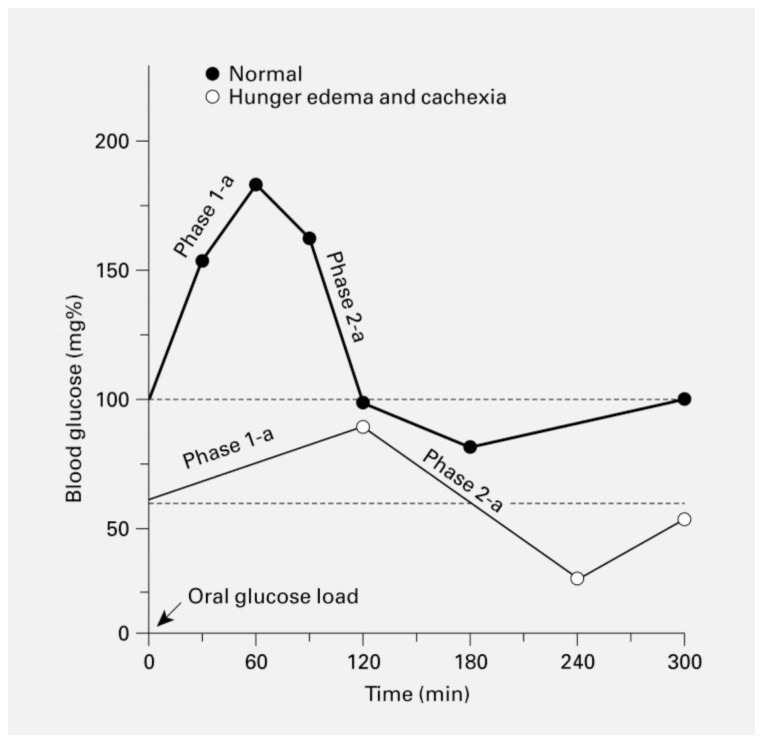
Glucose Tolerance Test Results in Normal Individuals and in Patients with Hunger Disease. Reproduction of the original figure in Winick^6^ by Massry and Smororzewski,^52^ with permission. Copyright © 2002, Karger Publishers, Basel, Switzerland.

The research performed in the Warsaw Ghetto led to some interesting findings regarding glucose metabolism in hunger disease. They reported that while the pancreas of hunger disease patients seemed to be normal, the response to glucose loading was abnormal (Figure 1).^6^ Fasting baseline glucose levels were lower than normal. This was attributed to lower metabolism as an adaptive mechanism to prolonged malnutrition. Following oral sugar loading, no glycosuria was observed in most of the patients. At the same time, blood glucose did not rise to the anticipated levels. The researchers hypothesized this to be secondary to either decreased intestinal absorption of glucose, or rapid trapping of glucose by “starved” organs such as the brain, heart, liver, and muscles. Initial increase in blood glucose levels was then followed by a period of hypoglycemia, which in some patients was profound. The sugar load given to their patients could be somewhat comparable with the candy that soldiers gave to the Bergen-Belsen survivors.^26^

Hence, in patients with limited physiologic reserves after prolonged starvation, it is plausible that hypoglycemia, metabolic imbalances such as hypophosphatemia, hypokalemia, and hypomagnesemia, along with an increased cardiac workload were precipitating factors to mortality. Specifically, they could have led to acute heart failure, decompensated cardiorespiratory failure, and the reported unexpected deaths.

## RELEVANCE FOR TODAY AND FINAL OBSERVATIONS

This review focused on lessons learned when faced with refeeding survivors of long-term mass starvation. Most of the more current literature, however, focuses on individual patients with associated pathologies at risk for refeeding syndrome and consists of reviews and case studies. Furthermore, most focus only on starvation as a complication of a medical or psychological condition, not the comprehensive clinical findings due to starvation, i.e. hunger disease.

Perhaps the most studied condition, with regard to refeeding syndrome, is anorexia nervosa. Like the Warsaw Ghetto cohort, patients with anorexia nervosa suffer from prolonged starvation, mostly without other medical comorbidities.^53^ Electrolyte imbalances such as hypophosphatemia, hypokalemia, and hypomagnesemia have been reported following refeeding of individuals suffering from anorexia nervosa.^54^,^55^ These electrolyte imbalances were also reported, mainly in case reports, in patients suffering from other mental health disorders,^56^ substance abuse,^57^ celiac disease,^58^ and cancer (particularly esophageal cancer).^59^ Refeeding syndrome has even been described in obese patients following bariatric surgery presenting with nutritional deficiencies.^60^ A recent case report found refeeding syndrome in a healthy individual who had been unknowingly starved—a reminder of the vigilance needed to diagnose the condition properly.^61^

All of the clinical conditions mentioned above have their own treatment protocols. It might therefore be possible for one to assume that different situations with varied deficiencies should handle refeeding differently. However, there is a huge difference between a patient being refed in a closely monitored hospital setting (and for whom a specific refeeding protocol might eventually be developed), and the circumstances of the studies explored in this review. The WW2 researchers were encountering thousands of malnourished victims of starvation. Most of their patients came from amongst these people, and many had already begun refeeding in an uncontrolled situation with no individual monitoring. This is a markedly different situation from that encountered today, upon which treatment guidelines were established and published in 2020.^53^

In clinical work, one often discovers similar previously published cases that have been forgotten. Indeed, the definition of refeeding syndrome that appeared by the end of the century was preceded by less publicized examples.^50^ The clinical studies performed in the Warsaw Ghetto exemplify overlooked yet critical research of relevance then and today. In light of the clandestine nature of their work, the researchers’ clinical conclusions are amazingly accurate and, if known earlier, could have helped save thousands of lives. It is highly unlikely that their research can ever again be repeated: it was performed on patients dying of starvation without any other known diseases.^6^ Their findings included metabolic and mineral imbalances and gave credence to descriptions of early death unrelated to underlying conditions.

Understanding this should help clinicians recognize that refeeding syndrome can potentially affect a much larger population group than just chronically ill individuals or those who have voluntarily starved themselves, be it for idealistic reasons or due to an eating disorder. According to the Action Against Hunger website, around 70% of the world’s refugees live in areas where food insecurity is an issue.^62^ Starvation is not far away from these people. This review has made it clear that their rescue and rehabilitation must take into consideration not just the risk of refeeding syndrome but the fact that any change in a patient’s status could trigger multiorgan failure and death. Hunger disease will most likely be encountered only in mass disaster situations, far removed from standard clinical practice. But given the current state of the world, it will be encountered. Clinicians must come to an understanding that hunger disease affects every aspect of the patient’s physiology. Hence, these patients will become incredibly difficult to manage, and incorrect refeeding could tip them over the edge. Awareness of these issues will help the stressed clinician better understand what is happening with their patients.

The basepoint for treating all patients at risk for refeeding syndrome, whatever the cause of their malnutrition, whatever their clinical presentation, remains the same. Winick, who worked with Columbia University to have the Warsaw Ghetto research translated into English, says it most succinctly: “… this study was not only the first to document the complex metabolic and circulatory changes that occur during semistarvation but the first to indicate the proper way to treat this condition. *Refeed these people slowly!*”^63^^(p3)^

## Abbreviations

POWs: prisoners of war
WW2: World War 2
